# Uptake Index of ^123^I-metaiodobenzylguanidine Myocardial Scintigraphy for Diagnosing Lewy Body Disease

**DOI:** 10.22038/aojnmb.2016.7972

**Published:** 2017

**Authors:** Yoshito Kamiya, Satoru Ota, Shintaro Okumiya, Kosuke Yamashita, Akihiro Takaki, Shigeki Ito

**Affiliations:** 1 Graduate School of Health Sciences, Kumamoto University, Kumamoto, Japan; 2 Fujifilm RI Pharma Co., Ltd., Tokyo, Japan; 3 Faculty of Life Sciences, Kumamoto University, Kumamoto, Japan; 4 Department of Medical Imaging, Faculty of Life Sciences, Kumamoto University, Kumamoto, Japan

**Keywords:** Iodine-123 metaiodobenzylguanidine, Lewy body disease, Quantification, Uptake index

## Abstract

**Objective(s):**

Iodine-123 metaiodobenzylguanidine (^123^I-MIBG) myocardial scintigraphy has been used to evaluate cardiac sympathetic denervation in Lewy body disease (LBD), including Parkinson’s disease (PD) and dementia with Lewy bodies (DLB). The heart-to-mediastinum ratio (H/M) in PD and DLB is significantly lower than that in Parkinson’s plus syndromes and Alzheimer’s disease. Although this ratio is useful for distinguishing LBD from non-LBD, it fluctuates depending on the system performance of the gamma cameras. Therefore, a new, simple quantification method using ^123^I-MIBG uptake analysis is required for clinical study. The purpose of this study was to develop a new uptake index with a simple protocol to determine ^123^I-MIBG uptake on planar images.

**Methods:**

The ^123^I-MIBG input function was obtained from the input counts of the pulmonary artery (PA), which were assessed by analyzing the PA time-activity curves. The heart region of interest used for determining the H/M was used for calculating the uptake index, which was obtained by dividing the heart count by the input count.

**Results:**

Forty-eight patients underwent ^123^I-MIBG chest angiography and planar imaging, after clinical feature assessment and tracer injection. The H/M and ^123^I-MIBG uptake index were calculated and correlated with clinical features. Values for LBD were significantly lower than those for non-LBD in all analyses (P<0.001). The overlapping ranges between non-LBD and LBD were 2.15 to 2.49 in the H/M method, and 1.04 to 1.22% in the uptake index method. The diagnostic accuracy of the uptake index (area under the curve (AUC), 0.98; sensitivity, 96%; specificity, 91%; positive predictive value (PPV), 90%; negative predictive value (NPV), 93%; and accuracy, 92%) was approximately equal to that of the H/M (AUC, 0.95; sensitivity, 93%; specificity, 91%; PPV, 90%; NPV, 93%; and accuracy, 92%) for discriminating patients with LBD and non-LBD.

**Conclusion:**

A simple uptake index method was developed using ^123^I-MIBG planar imaging and the input counts determined by analyzing chest radioisotope angiography images of the PA. The diagnostic accuracy of the uptake index was approximately equal to that of the H/M for discriminating patients with LBD and non-LBD.

## Introduction

Iodine-123 metaiodobenzylguanidine (^123^I-MIBG) has been used in cardiac sympathetic nerve imaging to assess heart failure, dilated cardiomyopathy, and cardiac arrhythmia (1-4). Furthermore, ^123^I-MIBG myocardial scintigraphy has been used to evaluate cardiac sympathetic denervation in Lewy body disease (LBD), including Parkinson’s disease (PD) and dementia with Lewy bodies (DLB) (5, 6). 

The heart-to-mediastinum ratio (H/M), determined using planar images, has been used for quantifying cardiac ^123^I-MIBG uptake (2, 7). The H/M in LBD is significantly lower than that in Parkinson’s plus syndromes and LBD from Alzheimer’s disease (AD) (5, 8). However, this method has limited accuracy because the H/M is obtained from two-dimensional image analysis, and is significantly affected by scatter from nearby organs and image acquisition parameters. Furthermore, the H/M fluctuates according to system performance (mainly collimator performance: low-energy, medium energy, low-medium energy, etc.) of the gamma cameras (9, 10). As a countermeasure, Nakajima et al. described the results of a large Japanese multicenter initiative (84 institutions), reporting cross-institution phantom calibrations for the measurement of the planar H/M by using standard nuclear cameras and collimators from a variety of vendors in order to obtain a standardized H/M (11, 12).

However, collimators with the same designation from different vendors have very different characteristics (13). Additionally, a complex experiment using a special phantom is required for calibrating system performance. Therefore, a new, simple method is required for quantifying cardiac ^123^I-MIBG uptake. Chen et al. and van der Veen et al. proposed that more accurate and reliable H/Ms may be obtained using single photon emission tomography (SPECT) imaging (14, 15). The results generated for the H/M are a relative uptake measure, and thus depend on the system performance of the gamma cameras. Previously, a quantification method using a retention index of the tracer in positron-emission tomography (PET) has been proposed (16, 17). The retention index was calculated using the ratio of arterial-blood counts and myocardial counts obtained using ^123^I-MIBG cadmium zinc telluride (CZT) SPECT image analysis (18). A simple and noninvasive image analysis technique without arterial blood sampling will also be useful in the clinical setting. Therefore, a simple, noninvasive quantification method using ^123^I-MIBG uptake image analysis is required for routine clinical study. 

We recently developed an I-123-*N*-isopropyl-*p*-iodoamphetamine (^123^I-IMP) microsphere method for quantifying cerebral blood flow by using chest dynamic planar images and brain SPECT (19). In addition, we developed a fully automated input function program by using the area under the curve (AUC) of the pulmonary artery (PA) and the integrated lung washout ratio (20). The AUC determination method for the PA could be applied for determining the input counts of ^123^I-MIBG (20). The output counts, as determined using the ^123^I-MIBG heart counts, could be analyzed using planar images.

The purpose of this study was to develop a new noninvasive quantification method with a simple protocol to determine ^123^I-MIBG uptake on planar images.

## Methods

### Theory

The input count obtained using the integrated counts of the time-activity curve (TAC) on the PA region of interest (ROI) of the chest radioisotope (RI) angiography images is directly proportional to the administration dose (20). Therefore, the uptake index can be noninvasively calculated as follows:

Uptake index=(output count)⁄(input count)×100 (%) (Eq. 1)

where output count (counts/pixel) is the heart count on the planar image.

The heart count (counts/pixel) was obtained by subtracting the background (BG) count (counts/pixel). The BG count was obtained by setting the ROI on the axillary dissection of the planar image.

**Figure 1:**
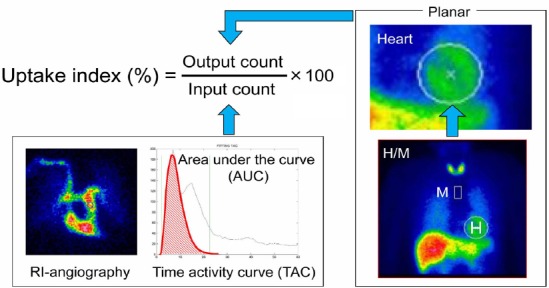
Region of interest (ROI) settings Input counts: Typical time-activity curves (TACs) from the pulmonary artery The first peak of the TAC is fitted with a gamma function, and the area under the count-time activity curve (AUC) is obtained by integrating the gamma functions Output counts: Heart counts from the conventional heart-to-mediastinum ratios

Figure 1 shows the scheme used for determining the uptake index. The first peak of the TAC was fitted with a gamma function, and the AUC was obtained by integrating the gamma functions. The heart ROI used for the H/M method was used for calculating the uptake index (Figure 1).

### Subjects

Table 1 lists the diseases of the patients who visited Chibana Clinic. Images collected from 48 patients (21 men, 27 women; age range: 56-89 years; mean age: 73.5 years) with clinically suspected LBD who underwent both ^123^I-MIBG chest RI angiography and planar imaging at the clinic between December 2013 and April 2014 were used for developing the theory and procedure for this new quantification method (Table 1). Clinical diagnosis was performed by a neurologist according to the neurological testing criteria for PD (21), DLB (22), and AD (23). Magnetic resonance imaging was used to rule out related diseases. None of the patients had cardiac disease, diabetes, and pulmonary disease. The age and sex were not significantly different between the patients in the two groups. 

**Table 1:** Classification and clinical diagnoses of 48 patients

Classification	LDB group (n=21)	non-LDB group (n=7)	*p*-value*
Sex (M/F)	8/13	13/14	0.836
Age	73.5±7.8 (57-89)	73.5±7.2 (56-84)	0.565
Clinical diagnosis	PD (n=16)	AD (n=5)	
	DLB (n=5)	VPS (n=7)	
		DPS(n=6)	
		Other(n=9)	

LBD, Lewy body disease; DLB, Dementia with Lewy bodies; PD, Parkinson's disease; AD, Alzheimer 's disease; VPS, Vascular parkinsonism; DPS, Drug-induced parkinsonism. Age data are presented as mean ± standard deviation (range).

* Fisher's exact test.

The study was approved by the Ethics Committee of Medicine at Chibana Clinic and the Kumamoto University for Human Studies, and written informed consent was obtained from all patients before the study began. All imaging data were handled anonymously, in accordance with the guidelines of the Declaration of Helsinki and the regulation of each institution’s ethics board.

### Study protocol

### Chest RI angiography

To determine the input function, ^123^I-MIBG chest RI angiography images in the anterior view were obtained for 2 min (1 s/frame, 128×128 matrix, and 3.3 mm/pixel) by using a detector equipped with low-medium-energy, general-purpose collimators after a bolus injection of 111 MBq of ^123^I-MIBG. The TAC of the PA was obtained by placing a circular ROI with a diameter of 3 pixels on the dynamic images of the PA (20).

### Planar imaging

To determine the output function, a chest planar image was acquired after dynamic image acquisition for 5 min (256×256 [1.45 zoom factor and 3.3 mm/pixel]), and the H/M and uptake index (%) on the planar image were calculated. The H/M was calculated using the method reported by Okuda et al. (7). The correlation between the H/M and uptake index was analyzed with respect to the observed clinical features.

### Statistical analysis

Statistically significant differences according to the patients’ sex were determined using Fisher’s exact test for binary variables. Continuous variables are presented as mean ± standard deviation and median. Where indicated, intergroup differences were determined using a two-sided Student’s t-test or the Mann-Whitney U test. The diagnostic values of the H/M and uptake index methods were assessed by calculating the area under the receiver-operating characteristic (ROC) curve. Diagnostic accuracy was evaluated by calculating sensitivity, specificity, positive predictive value (PPV), and negative predictive value (NPV). The criterion value of each method was based on the Youden index (J) of the ROC curve. Analyses were performed using MedCalc software (version 12.4). Statistical significance for all analyses was assessed at a probability value of less than 0.05.

## Results

**Figure 2:**
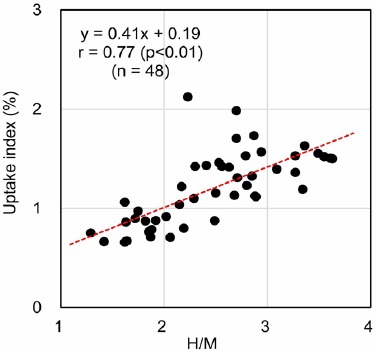
Correlation between the heart-to-mediastinum ratio (H/M) and uptake indexer the curve)

Figure 2 shows the linear regression analysis between the H/M and uptake index methods. Both values, obtained by these different techniques, were found to be correlated (r=0.77, P<0.001, n=48).

**Figure 3:**
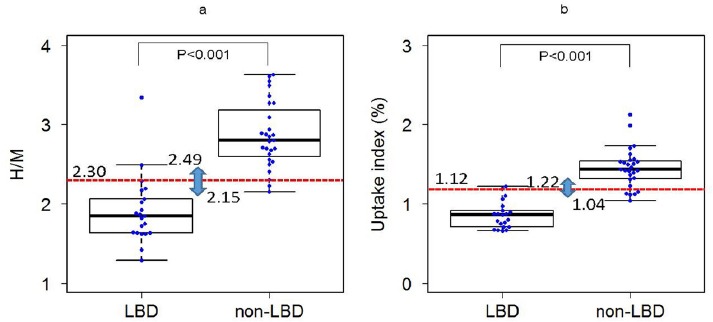
Box-and-whisker plots comparing the patients with Lewy body disease (LBD) and those with non-LBD The probability value indicates the significance level for two groups by using the Mann-Whitney U test. The red line indicates the optimal cutoff value determined using the receiver-operating characteristic (ROC) analysis Lower fence = Q_1_ - (1.5×IQR) and Upper fence = Q_3_ + (1.5×IQR), where Q_1_ and Q_3_ are the first and third quartiles, and IQR is the interquartile range a. Box-and-whisker plots of the heart-to-mediastinum ratio (H/M) (cutoff value, 2.30) b. Box-and-whisker plots of the uptake index (cutoff value, 1.12%)

Figure 3 shows box-and-whiskers plots comparing the LBD and non-LBD patients in the H/M and uptake index. The H/M values of the LBD in ten patients were lower than that of the non-LBD patients (P<0.01). The H/M values of the LBD patients were found to range from 1.29 to 3.34 (mean: 1.92, median: 1.85, interquartile range (IQR): 1.29-2.06, variance: 0.190, 95% CI: 1.72-2.11) and that of the non-LBD patients were within the range of 2.15-3.63 (mean: 2.87, median: 2.80, IQR: 2.60-3.18, variance: 0.176, 95% CI: 2.70-3.03) (Figure 3a). The uptake values of the LBD patients were found to range from 0.66 to 1.22 (mean: 0.86, median: 0.86, IQR: 0.71-0.92, variance: 0.029, 95% CI: 0.78-0.94) and that of the non-LBD patients were within the range of 1.04-2.12 (mean: 1.45, median: 1.43, IQR: 1.32-1.54, variance: 0.063, 95% CI: 1.35-1.55) (Figure 3b). The overlapped ranges between non-LBD and LBD were 2.15 to 2.49 in the H/M method, and 1.04 to 1.22% in the uptake index method. The optimal cutoff values of the H/M and uptake index were 2.30, and 1.12, respectively.

**Figure 4:**
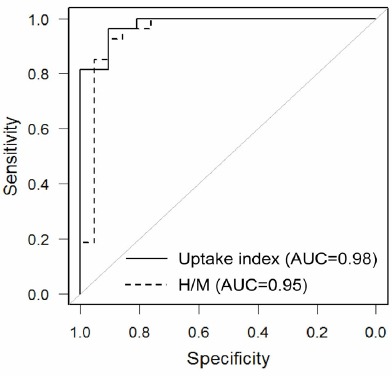
Receiver-operating characteristic (ROC) curves (Dotted line, heart-to-mediastinum ratio [H/M] (AUC=0.95); Solid line, uptake index (AUC=0.98); AUC, area und

Figure 4 and Table 2 compare ROC curves and diagnostic accuracy of the H/M and uptake index. The diagnostic accuracy of the uptake index (AUC 0.98, sensitivity 96%, specificity 91%, PPV 90%, NPV 93%, accuracy 92%) was approximately equal to the H/M (AUC 0.95, sensitivity 93%, specificity 91%, PPV 90%, NPV 93%, accuracy 92%) for discrimination of the LBD and non-LBD patients.

**Table 2:** Diagnostic performance of two variables in the Lewy body disease (LBD) and non-LBD groups

Method	AUC	Sensitivity	Specificity	Cutoff	95% Cl	PPV*	NPV^†^	Accuracy
Planar H/M	0.95	0.93	0.91	<2.30	0.87 - 1	0.90	0.93	0.92

Planar uptake (%)	0.98	0.96	0.91	<1.12	0.95 - 1	0.90	0.93	0.92

AUC, area under the curve; 95% CI, 95 percent confidence interval.

* PPV, positive predictive value;

† NPV, negative predictive value.

H/M, heart to mediastinum ratio.

## Discussion

By analyzing chest RI angiography images, we have established a new uptake index method for ^123^I-MIBG heart uptake measurements. The input count was calculated by using the integrated counts of the TAC on the PA ROI, by modifying the simple non-invasive ^123^I-IMP quantifition based on a microsphere model according to the pharmacokinetics of ^123^I-MIBG (19, 20). The heart count was determined using the same method as the H/M.

The results and diagnostic accuracy of the uptake index were approximately equal to those of the H/M. The H/M values depend on the system performance of the gamma cameras because of the low count fluctuations of the mediastinum as BG counts (13). Nakajima et al. have tried to standardize the H/M values at multiple facilities in order to reduce this fluctuation (24). Standardization of the H/M method requires the use of a performance conversion coefficient according to system performance. Therefore, a special experiment will be required for calibrating the system in order to obtain the performance conversion coefficient.

Generally, the heart uptake value is obtained by dividing the heart activity (Bq) by the input activity (Bq). When the planar heart and input counts were obtained by one system, the uptake value is calculated without an experimental count-activity conversion calculation (CC), because the CC is offset. Therefore, the uptake value does not depend on the system performance of the gamma cameras. This is an advantage of the uptake index method.

The results of this study were obtained from patients by using a single gamma camera. Further study is necessary to confirm the feasibility of this method across multiple facilities. The validity and accuracy would ideally need to be confirmed using planar images in a different patient group.

The input count analysis involves complex techniques. Recently, we developed an automated, simple, and noninvasive ^123^I-IMP microsphere input function determination program (auto-SIMS program). This program consisted of two ROI setting programs for the PA and lung regions that used the image phase analysis of a chest RI angiogram (20). An automated uptake index program can be easily developed by modifying the PA ROI setting algorithm of the auto-SIMS program. If this program could be developed, subsequent analysis of the uptake index could be completed with one click in a few seconds, without the need for performing complex analyses. In addition, this automated program will improve the repeatability and reproducibility of this uptake method. When the automated program for the uptake index method is completed, this method could be applied as a useful and stable method in routine clinical studies.

Giorgetti et al. reported a quantification method using ^123^I-MIBG SPECT with a CZT camera (18). If the uptake index method using ^123^I-MIBG SPECT could be developed by referring to their method, its diagnostic accuracy could be improved, such that its diagnostic accuracy was higher than that of the H/M method.

## Conclusion

We developed a simple uptake index method by using ^123^I-MIBG planar heart and input counts determined by analyzing chest RI angiography images. The diagnostic accuracy of the uptake index was approximately equal to that of the H/M for discriminating patients with LBD and non-LBD. Further testing is necessary to confirm the feasibility of this method in other facilities.
